# Supplementary figures and images for: Antigen-specific T cell responses following single and co-administration of tick-borne encephalitis, Japanese encephalitis, and yellow fever virus vaccines: Results from an open-label, non-randomized clinical trial-cohort
Source: PLoS Negl Trop Dis. 2025 Feb 28;19(2):e0012693. doi: 10.1371/journal.pntd.0012693 (PMC11893121; doi:10.1371/journal.pntd.0012693)

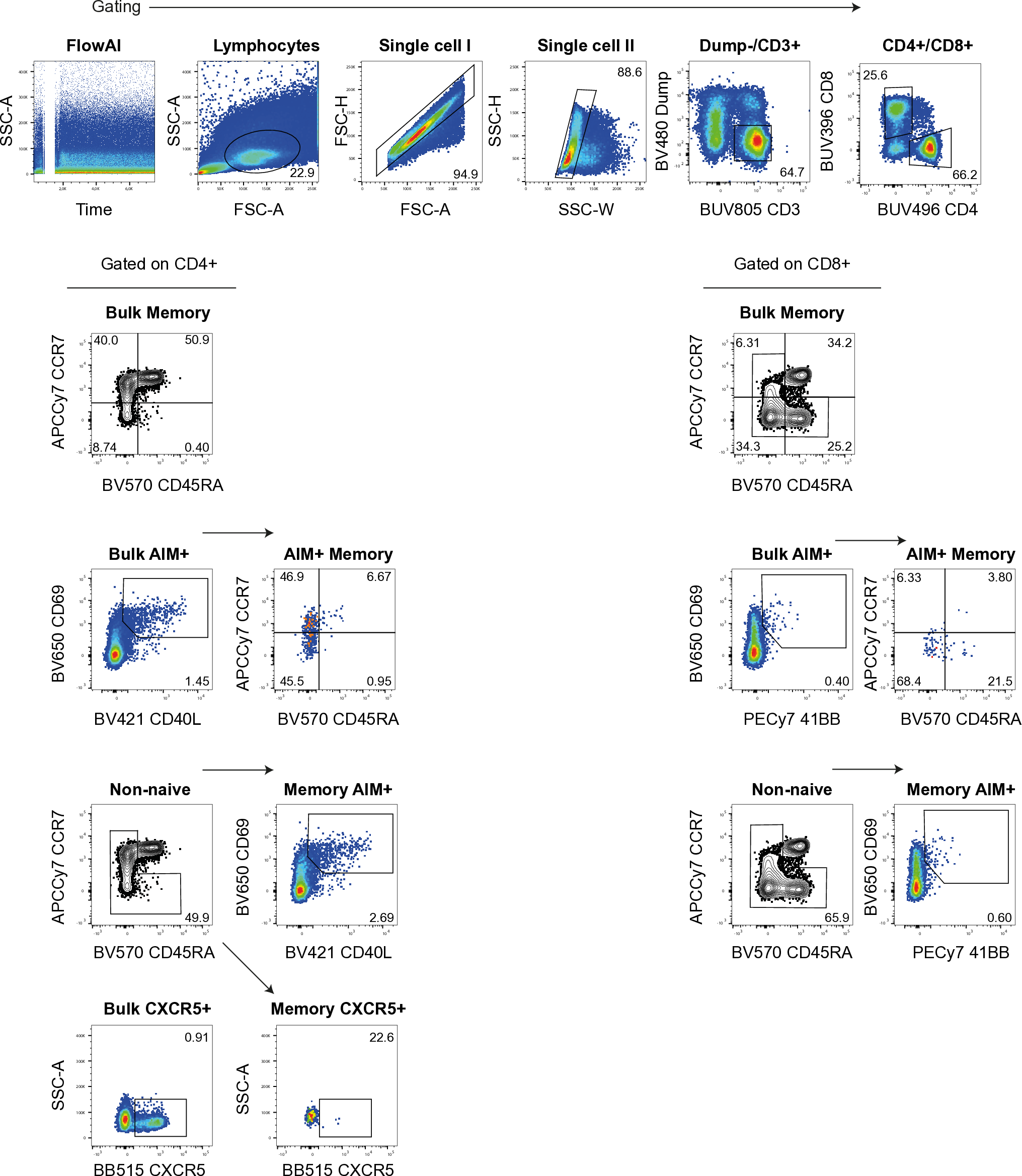

Supplement: S1 Fig — Lymphocytes were gated followed by subsequent singlet gating. Cells were then gated as CD3+ and Dump- (dead cells, CD19+ and CD14+). T cells were subdivided as either CD4+ or CD8+ cells. CD4+ T follicular helper cells were identified as CXCR5+. CD4+ and CD8+ (bulk) memory cell phenotypes were defined by CD45RA and CCR7 expression. CD4+ and CD8+ AIM+ cells were gated on bulk and non-naive cells (CCR7+CD45RA+). Antigen-specific T cells were identified with AIM markers, where antigen-specific CD4+ T cells were defined by CD69+CD40L+ and antigen-specific CD8+ T cells as CD69+41BB+. (TIF) [file pntd.0012693.s001.tif]

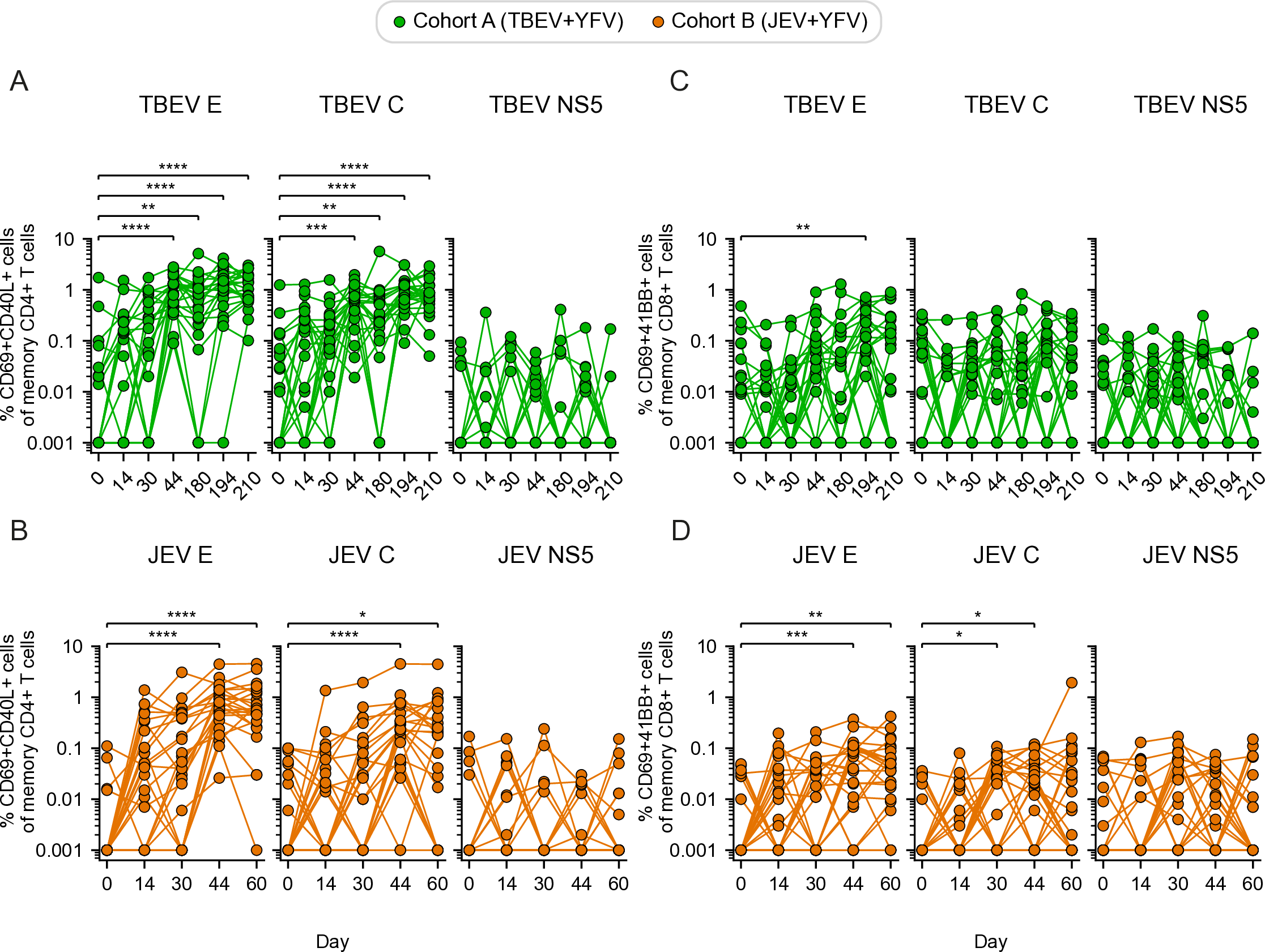

Supplement: S2 Fig — (A-B) Frequency of TBEV- and JEV-specific E, C, and NS5 CD4+ T cells in the (A) TBEV+YFV (cohort A) and (B) JEV+YFV (cohort B), respectively. (C-D) Frequency of TBEV- and JEV-specific E, C, and NS5 CD8+ T cells in the (C) TBEV+YFV (cohort A) and (D) JEV+YFV (cohort B), respectively. (A-D) Statistical analysis assessed by Friedman test, with Siegel and Castellan’s All-Pairs Comparisons post hoc tests with Bonferroni correction. Significant results are shown, where: *p ≤ 0.05, **p ≤ 0.01, ***p ≤ 0.001, ****p ≤ 0.0001. (TIF) [file pntd.0012693.s002.tif]

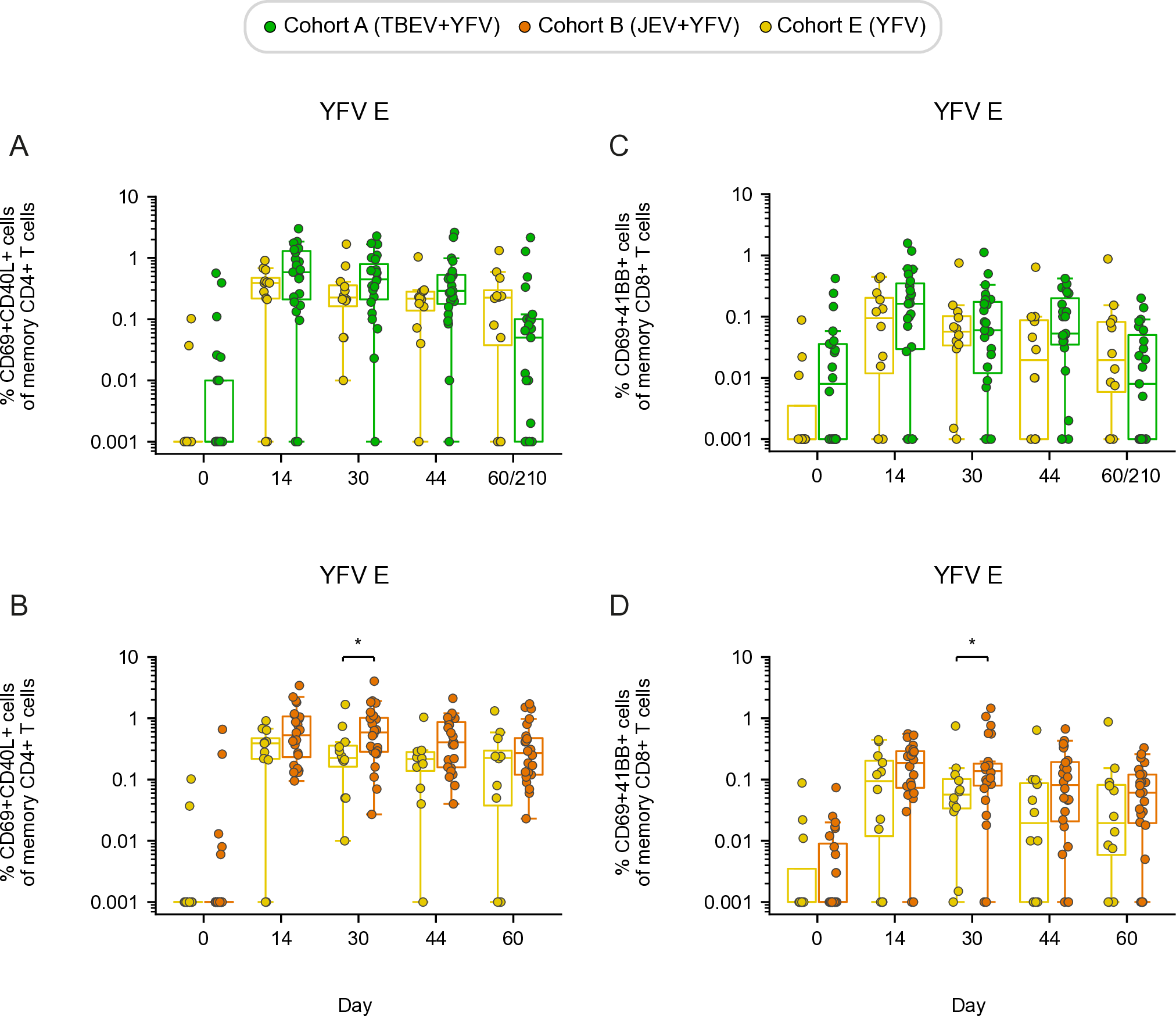

Supplement: S3 Fig — (A) Frequency of YFV E-specific CD4+ T cells between study participants from cohort E (YFV) and cohort A (TBEV+YFV) and (B) frequency of YFV E-specific CD4+ T cells between study participants from cohort E and cohort B (JEV+YFV). (C) Frequency of YFV E-specific CD8+ T cells between study participants from cohort E and cohort A and (D) frequency of YFV E-specific CD8+ T cells between cohort E and cohort B. (A-D) Statistical analysis assessed by Mann-Whitney rank test at each time point. Distribution of data points is determined by boxplot with median and 25th-75th percentiles; whiskers are drawn from 1.5 times the IQR. Significant results are shown, where: *p ≤ 0.05, **p ≤ 0.01, ***p ≤ 0.001, ****p ≤ 0.0001. (TIF) [file pntd.0012693.s003.tif]

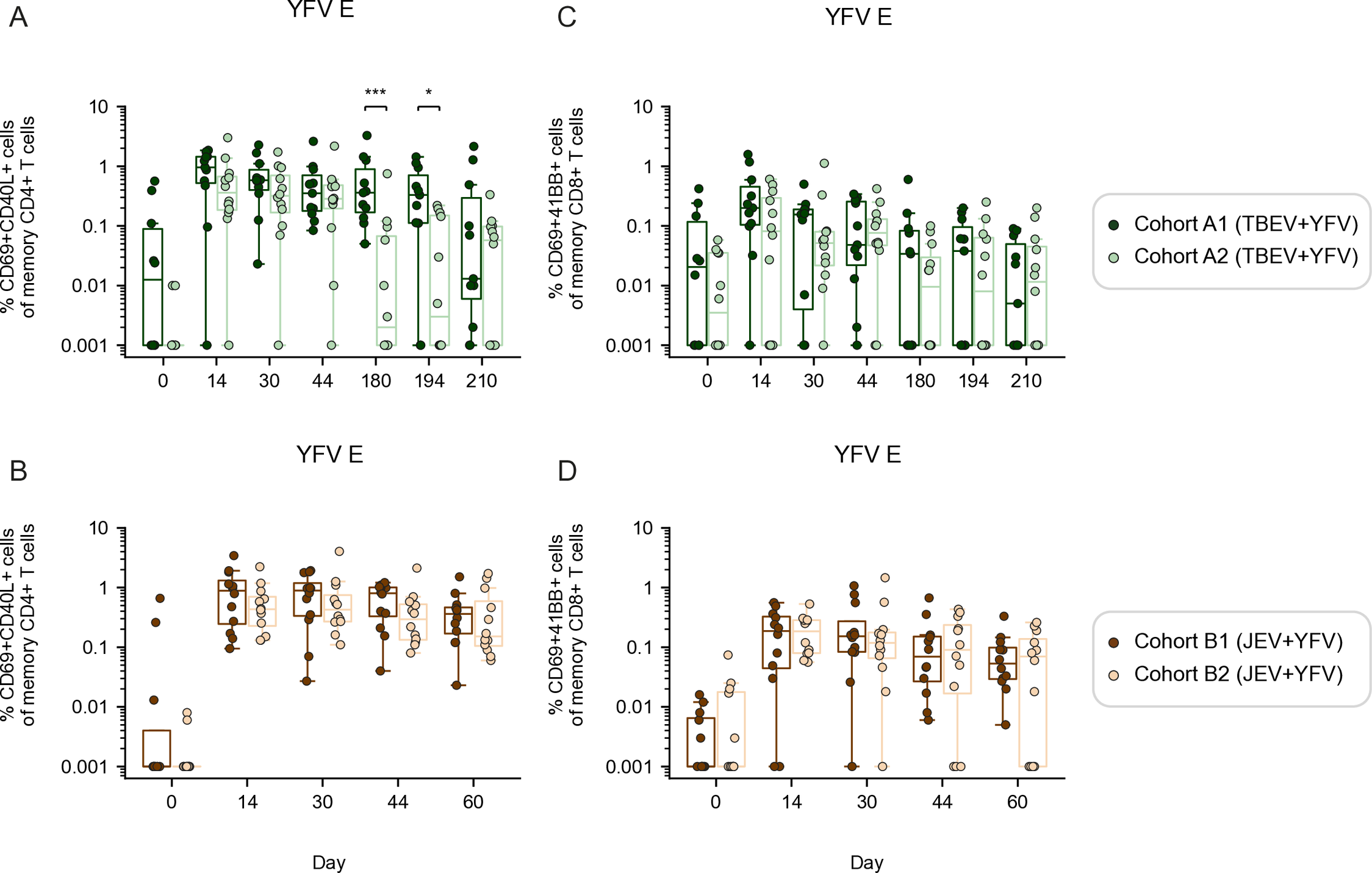

Supplement: S4 Fig — (A) Frequency of YFV E-specific CD4+ T cells compared between cohort A1 and A2. (B) Frequency of YFV E-specific CD4+ T cells compared between cohort B1 and B2. (C) Frequency of YFV E-specific CD8+ T cells compared between cohort A1 and A2. (D) Frequency of YFV E-specific CD8+ T cells compared between cohort B1 and B2. (A-D) Statistical analysis assessed by Mann-Whitney rank test at each time point. Distribution of data points is determined by boxplot with median and 25th-75th percentiles; whiskers are drawn from 1.5 times the IQR. Significant results are shown, where: *p ≤ 0.05, **p ≤ 0.01, ***p ≤ 0.001, ****p ≤ 0.0001. (TIF) [file pntd.0012693.s004.tif]

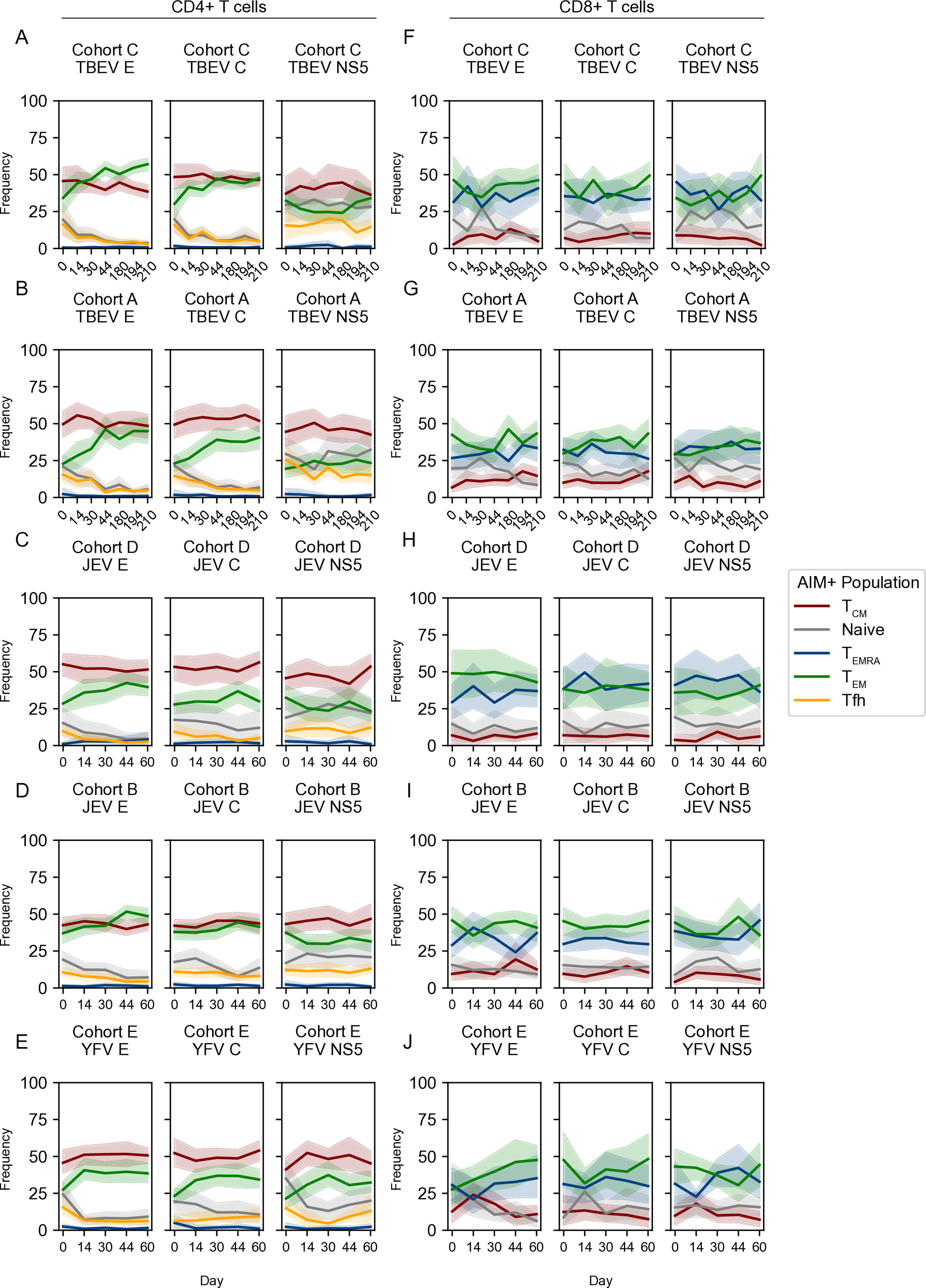

Supplement: S5 Fig — (A-E) Frequencies of TEM, TCM, TEMRA, naive and Tfh of CD69+CD40L+ among CD4+ T cells for cohort C, A, D, B and E, respectively. (F-J) Frequencies of TEM, TCM, TEMRA, and naive of CD69+41BB+ among CD8+ T cells for cohort C, A, D, B and E, respectively. (TIF) [file pntd.0012693.s005.tif]

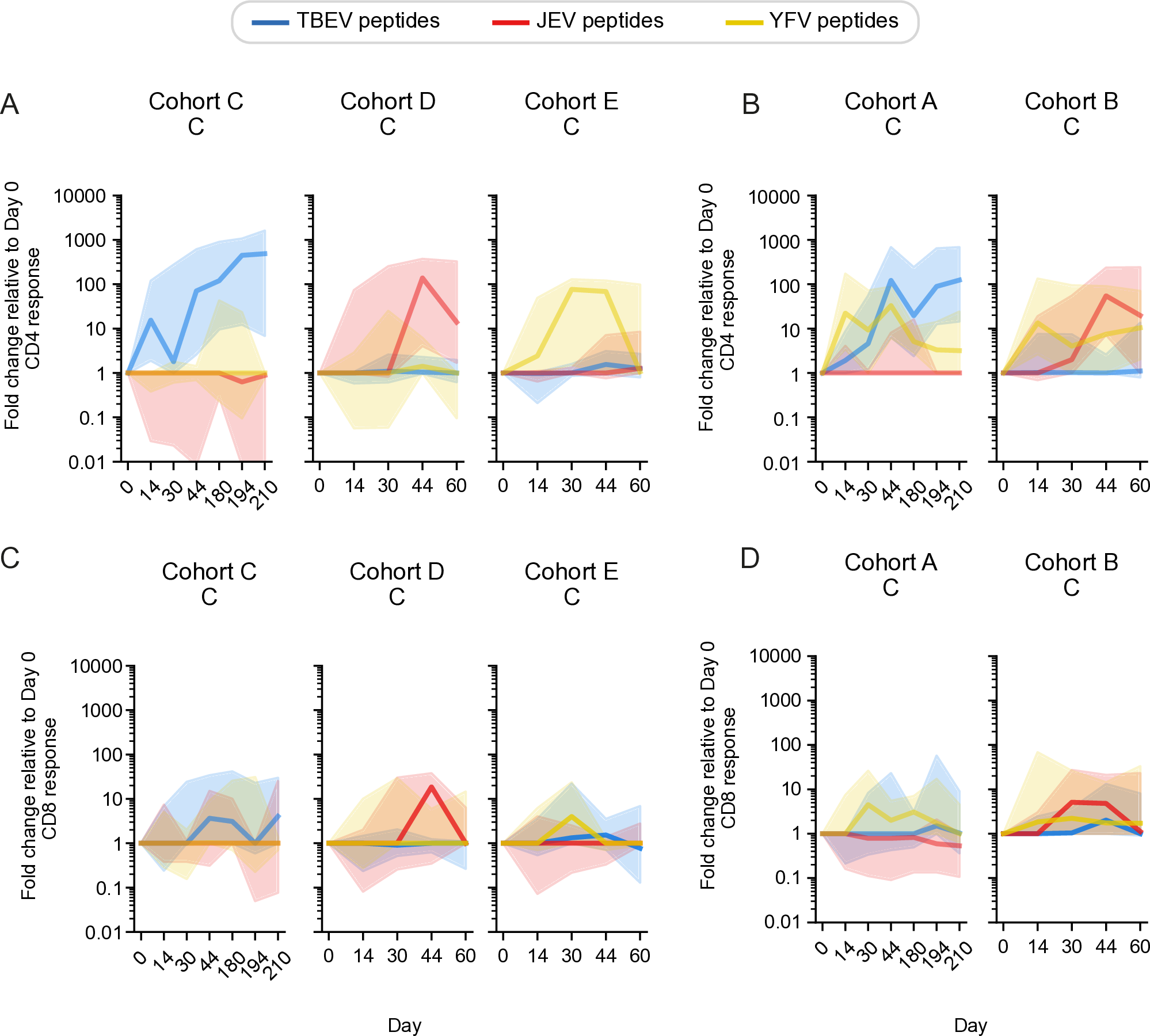

Supplement: S6 Fig — Crossreactive C-specific T cell responses in the vaccine cohorts. (A-B) Frequency of TBEV, JEV, and YFV C-specific CD4+ T cells and (C-D) TBEV, JEV, and YFV C-specific CD8+ T cells in study participants from all cohorts expressed as fold change relative to day 0. Line plots show median and 95% confidence interval (shaded area). (TIF) [file pntd.0012693.s006.tif]

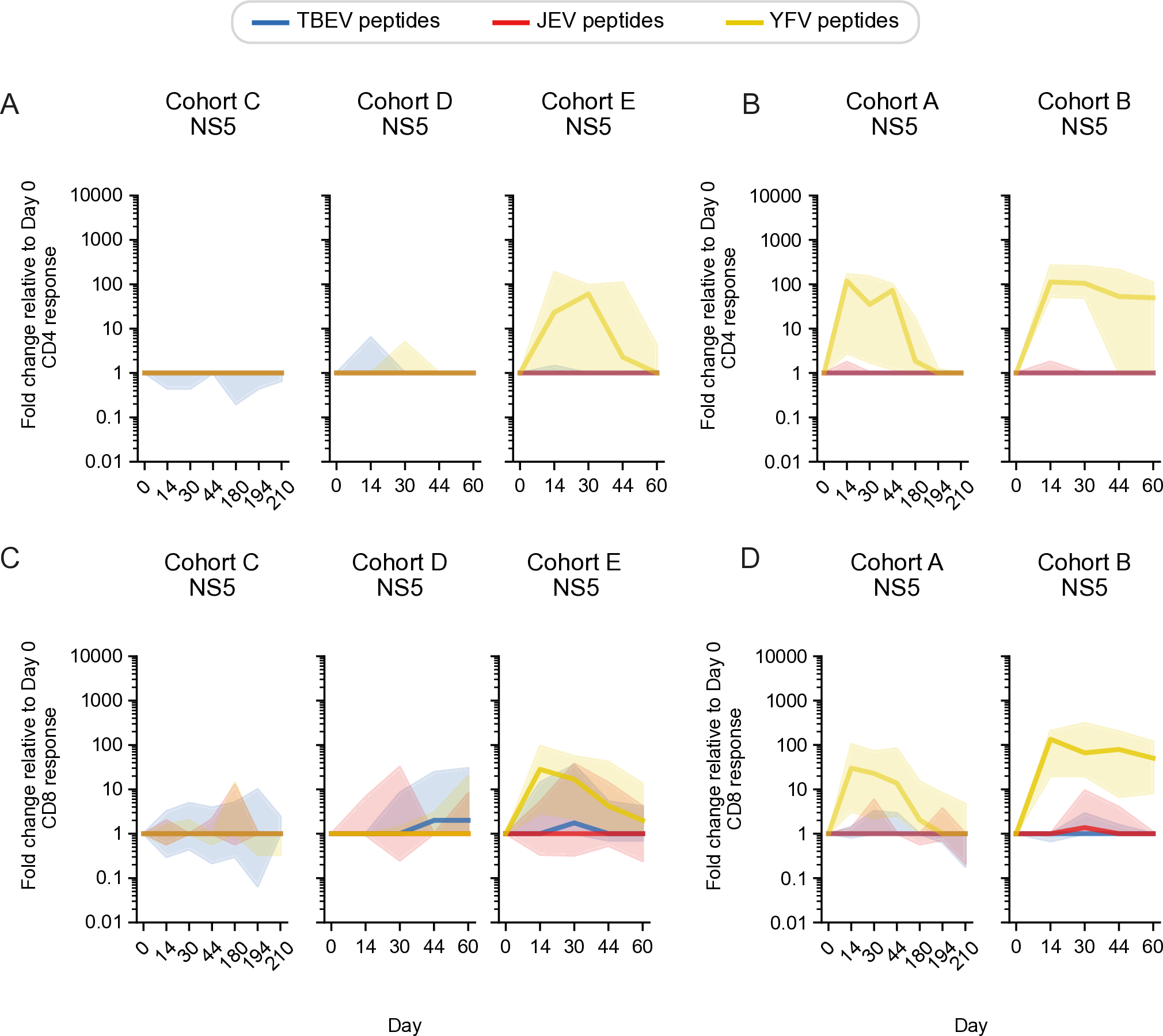

Supplement: S7 Fig — Crossreactive NS5-specific T cell responses in the vaccine cohorts. (A-B) Frequency of TBEV, JEV, and YFV (NS5-specific CD4+ T cells and (C-D) TBEV, JEV, and YFV NS5-specific CD8+ T cells in study participants from all cohorts expressed as fold change relative to day 0. Line plots show median and 95% confidence interval (shaded area). (TIF) [file pntd.0012693.s007.tif]

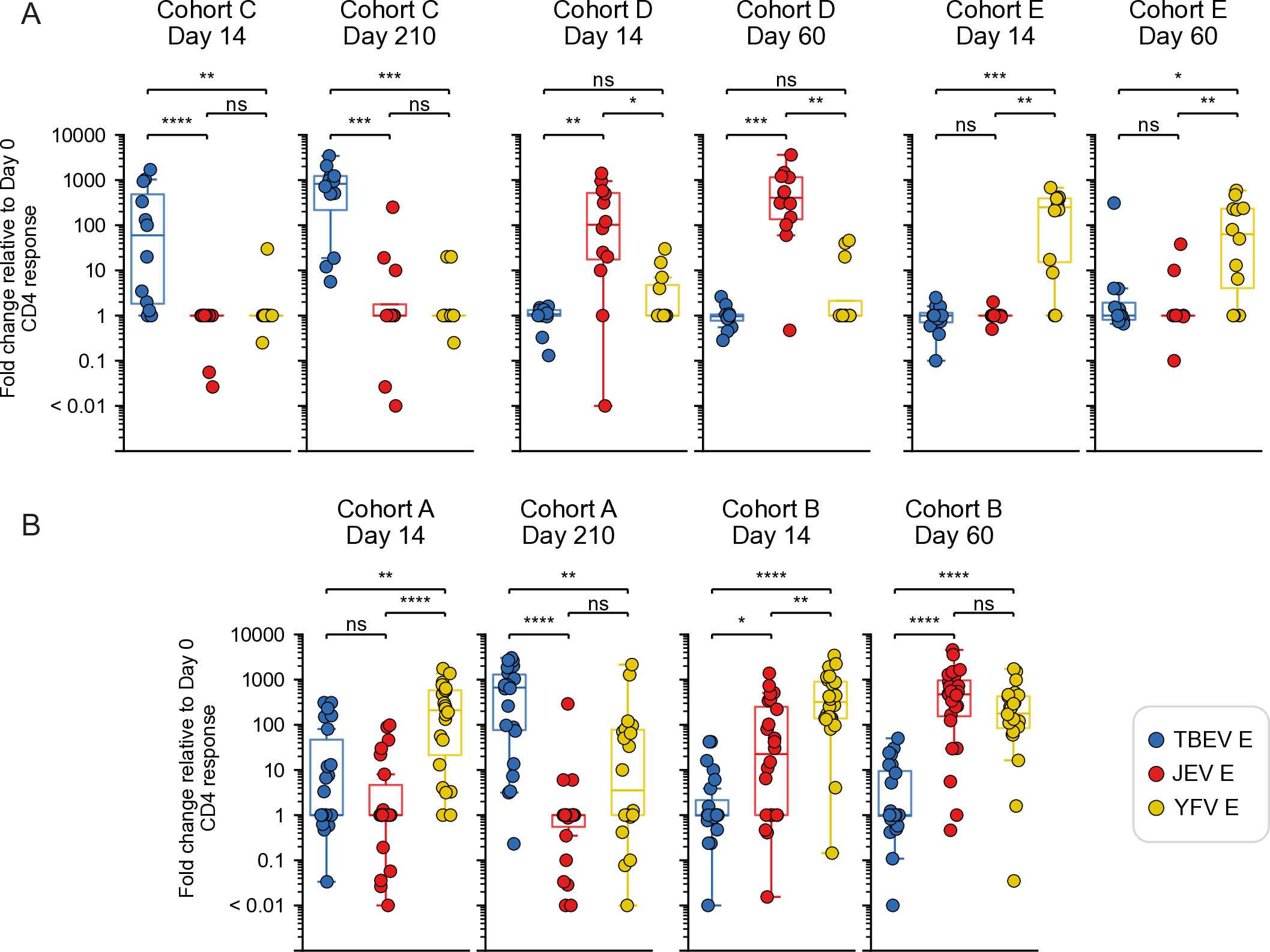

Supplement: S8 Fig — Comparisons of cross-reactive E-specific CD4+ T cell responses in the vaccine cohorts. (A-B) Frequency of TBEV E-specific CD4+ T cells in (A) cohorts C, D and E and (B) in cohorts A and B, expressed as fold change relative to day 0. (A-B) Statistical analysis assessed by Kruskal-Wallis’s and post hoc Dunn’s tests with Bonferroni correction. Distribution of data points is determined by boxplot with median and 25th-75th percentiles; whiskers are drawn from 1.5 times the IQR. Significant results are shown, where: *p ≤ 0.05, **p ≤ 0.01, ***p ≤ 0.001, ****p ≤ 0.0001. (TIF) [file pntd.0012693.s008.tif]

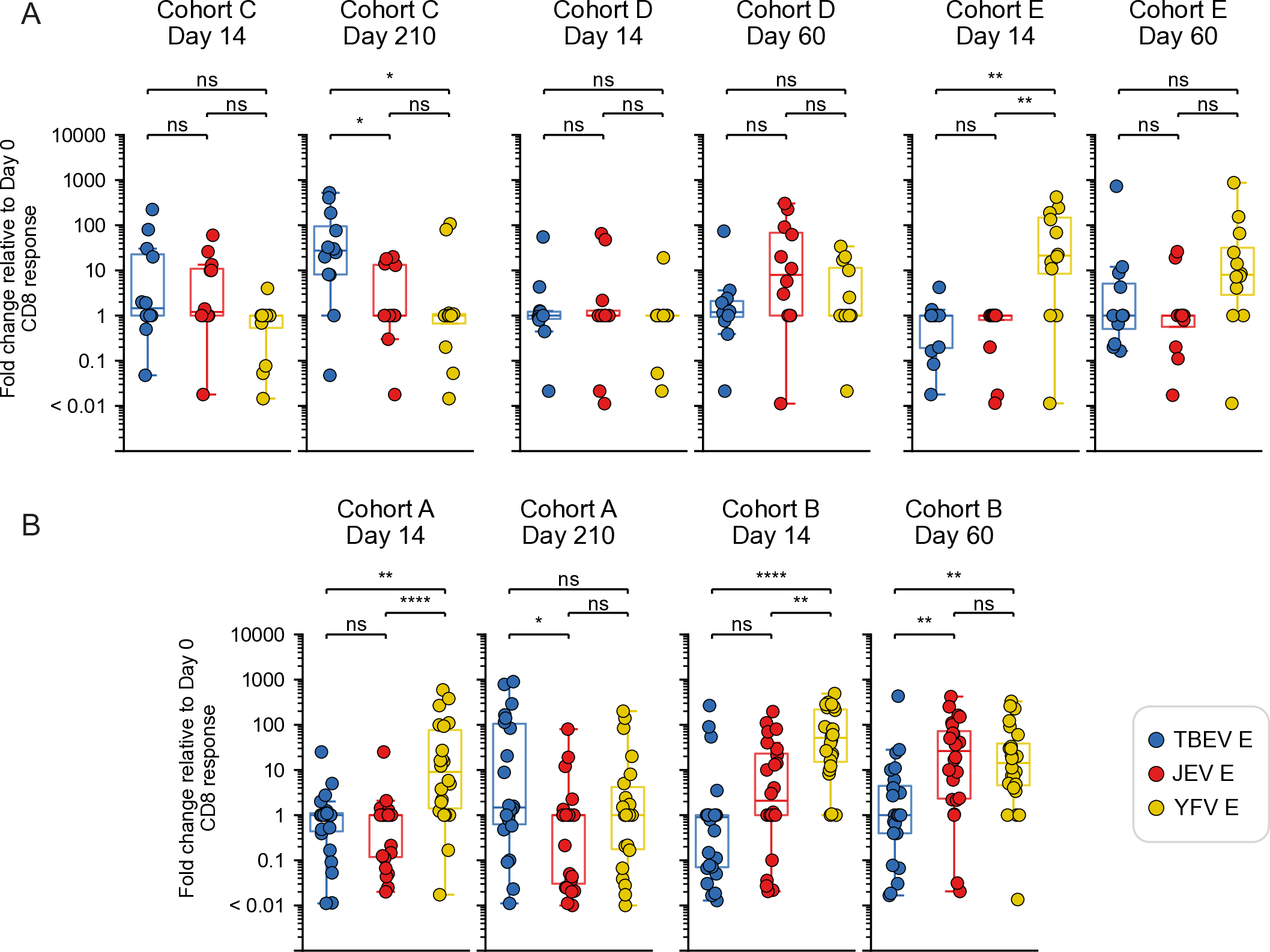

Supplement: S9 Fig — Comparisons of cross-reactive E-specific CD8+ T cell responses in the vaccine cohorts. (A-B) Frequency of TBEV E-specific CD8+ T cells in (A) cohorts C, D and E and (B) in cohorts A and B, expressed as fold change relative to day 0. (A-B) Statistical analysis assessed by Kruskal-Wallis’s and post hoc Dunn’s tests with Bonferroni correction. Distribution of data points is determined by boxplot with median and 25th-75th percentiles; whiskers are drawn from 1.5 times the IQR. Significant results are shown, where: *p ≤ 0.05, **p ≤ 0.01, ***p ≤ 0.001, ****p ≤ 0.0001. (TIF) [file pntd.0012693.s009.tif]

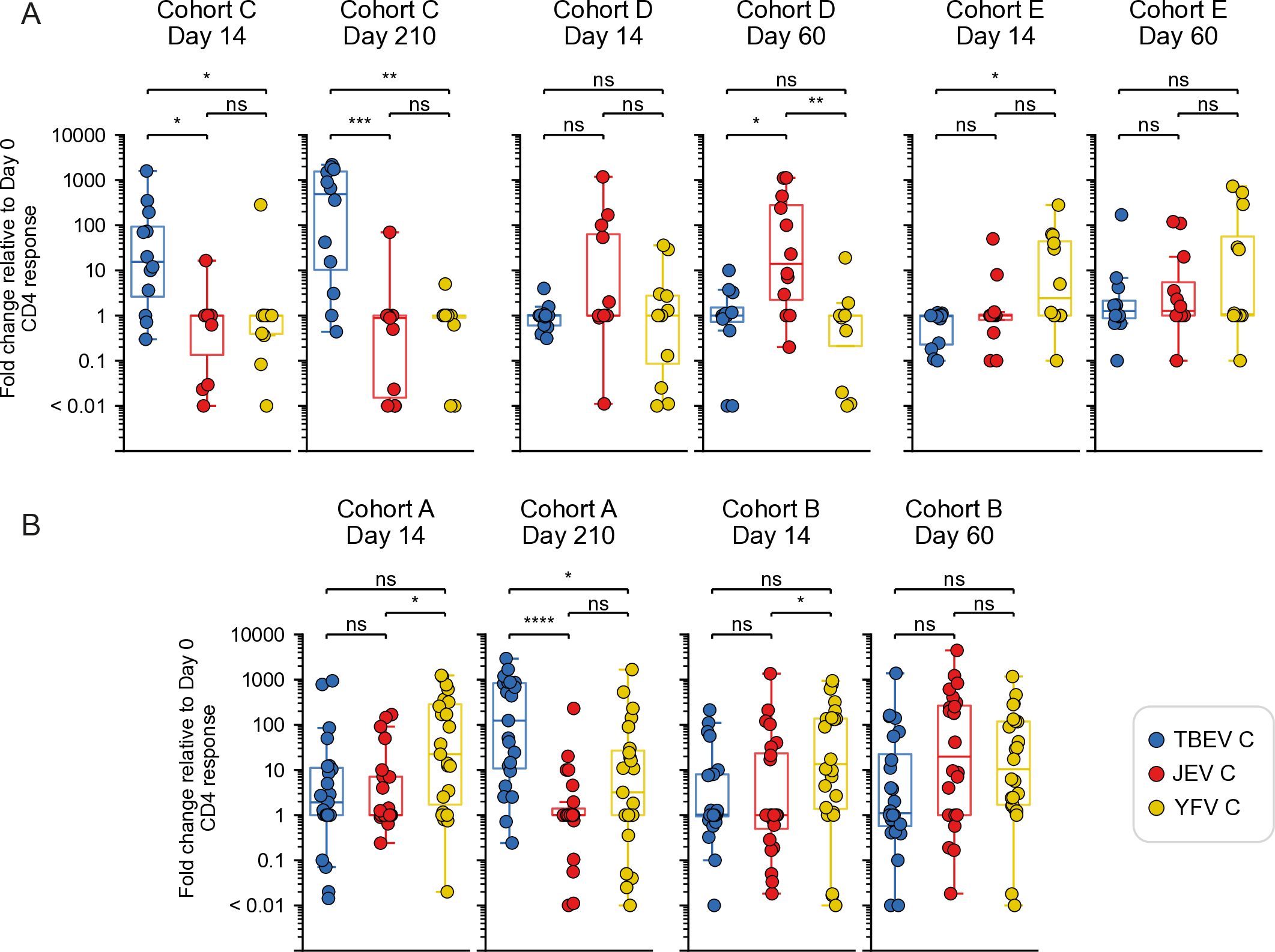

Supplement: S10 Fig — Comparisons of cross-reactive C-specific CD4+ T cell responses in the vaccine cohorts. (A-B) Frequency of TBEV C-specific CD4+ T cells in (A) cohorts C, D and E and (B) in cohorts A and B, expressed as fold change relative to day 0. (A-B) Statistical analysis assessed by Kruskal-Wallis’s and post hoc Dunn’s tests with Bonferroni correction. Distribution of data points is determined by boxplot with median and 25th-75th percentiles; whiskers are drawn from 1.5 times the IQR. Significant results are shown, where: *p ≤ 0.05, **p ≤ 0.01, ***p ≤ 0.001, ****p ≤ 0.0001. (TIF) [file pntd.0012693.s010.tif]

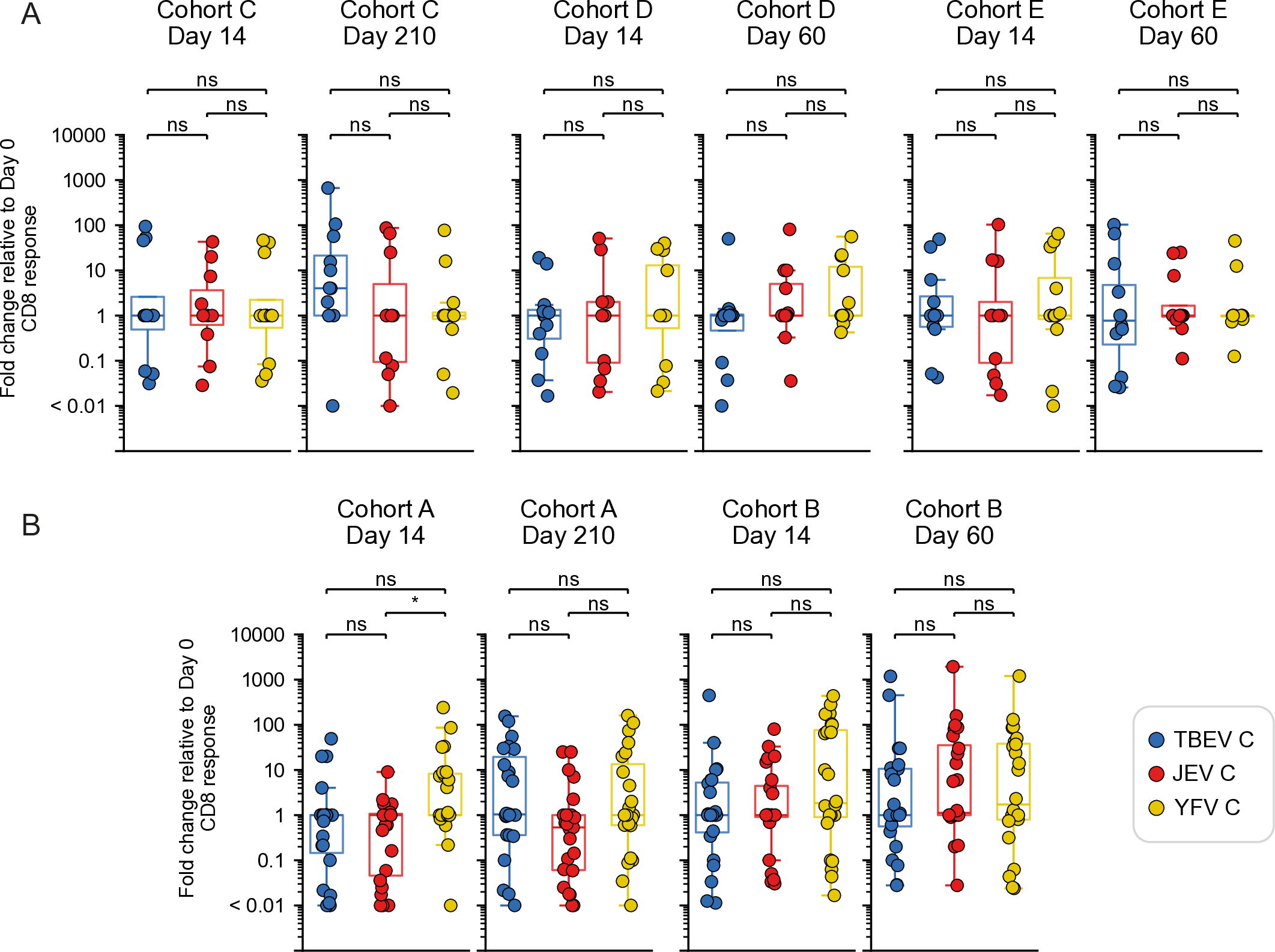

Supplement: S11 Fig — Comparisons of cross-reactive C-specific CD8+ T cell responses in the vaccine cohorts. (A-B) Frequency of TBEV C-specific CD8+ T cells in (A) cohorts C, D and E and (B) in cohorts A and B, expressed as fold change relative to day 0. (A-B) Statistical analysis assessed by Kruskal-Wallis’s and post hoc Dunn’s tests with Bonferroni correction. Distribution of data points is determined by boxplot with median and 25th-75th percentiles; whiskers are drawn from 1.5 times the IQR. Significant results are shown, where: *p ≤ 0.05, **p ≤ 0.01, ***p ≤ 0.001, ****p ≤ 0.0001. (TIF) [file pntd.0012693.s011.tif]

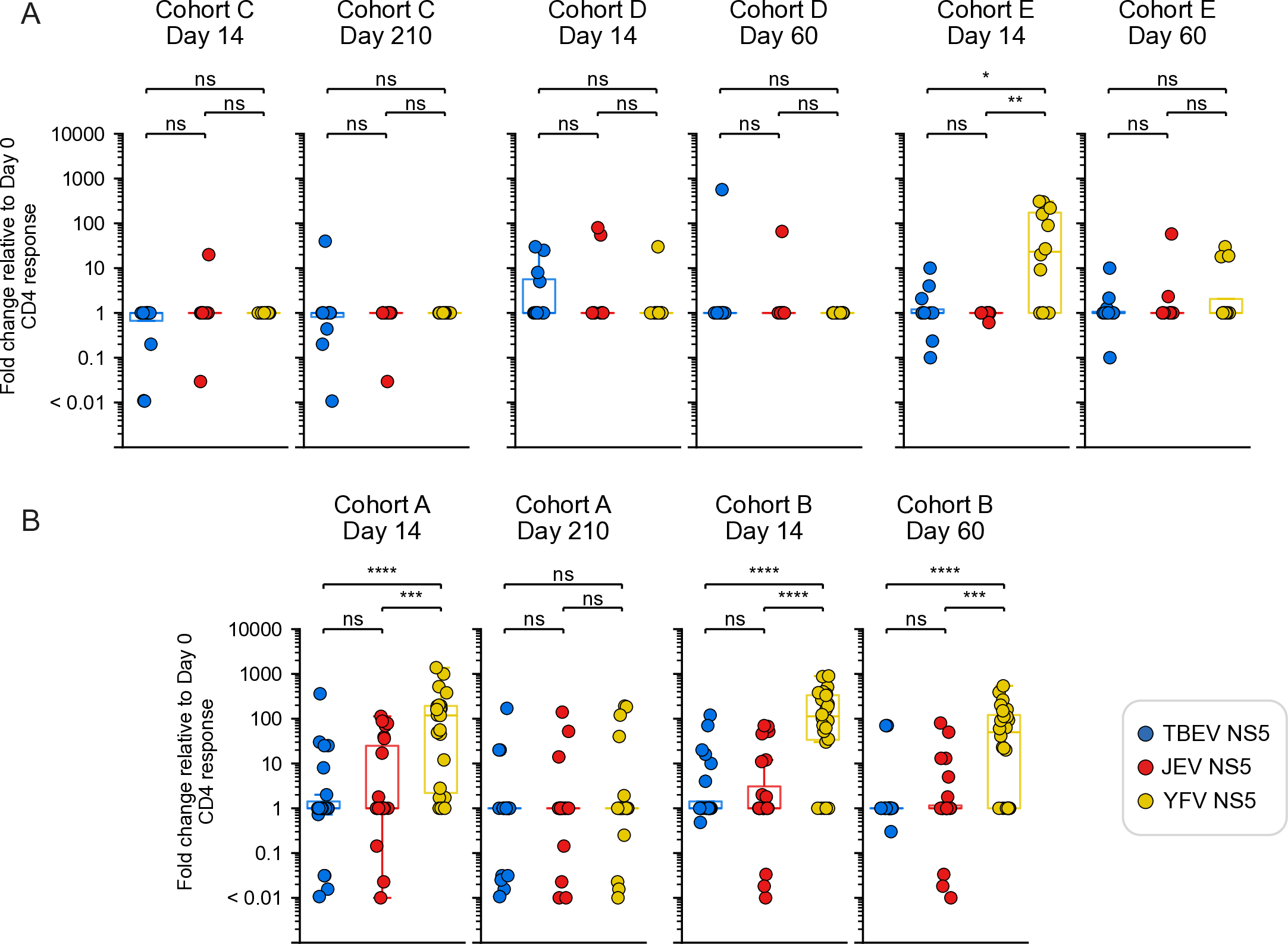

Supplement: S12 Fig — Comparisons of cross-reactive NS5-specific CD4+ T cell responses in the vaccine cohorts. (A-B) Frequency of TBEV NS5-specific CD4+ T cells in (A) cohorts C, D and E and (B) in cohorts A and B, expressed as fold change relative to day 0. (A-B) Statistical analysis assessed by Kruskal-Wallis’s and post hoc Dunn’s tests with Bonferroni correction. Distribution of data points is determined by boxplot with median and 25th-75th percentiles; whiskers are drawn from 1.5 times the IQR. Significant results are shown, where: *p ≤ 0.05, **p ≤ 0.01, ***p ≤ 0.001, ****p ≤ 0.0001. (TIF) [file pntd.0012693.s012.tif]

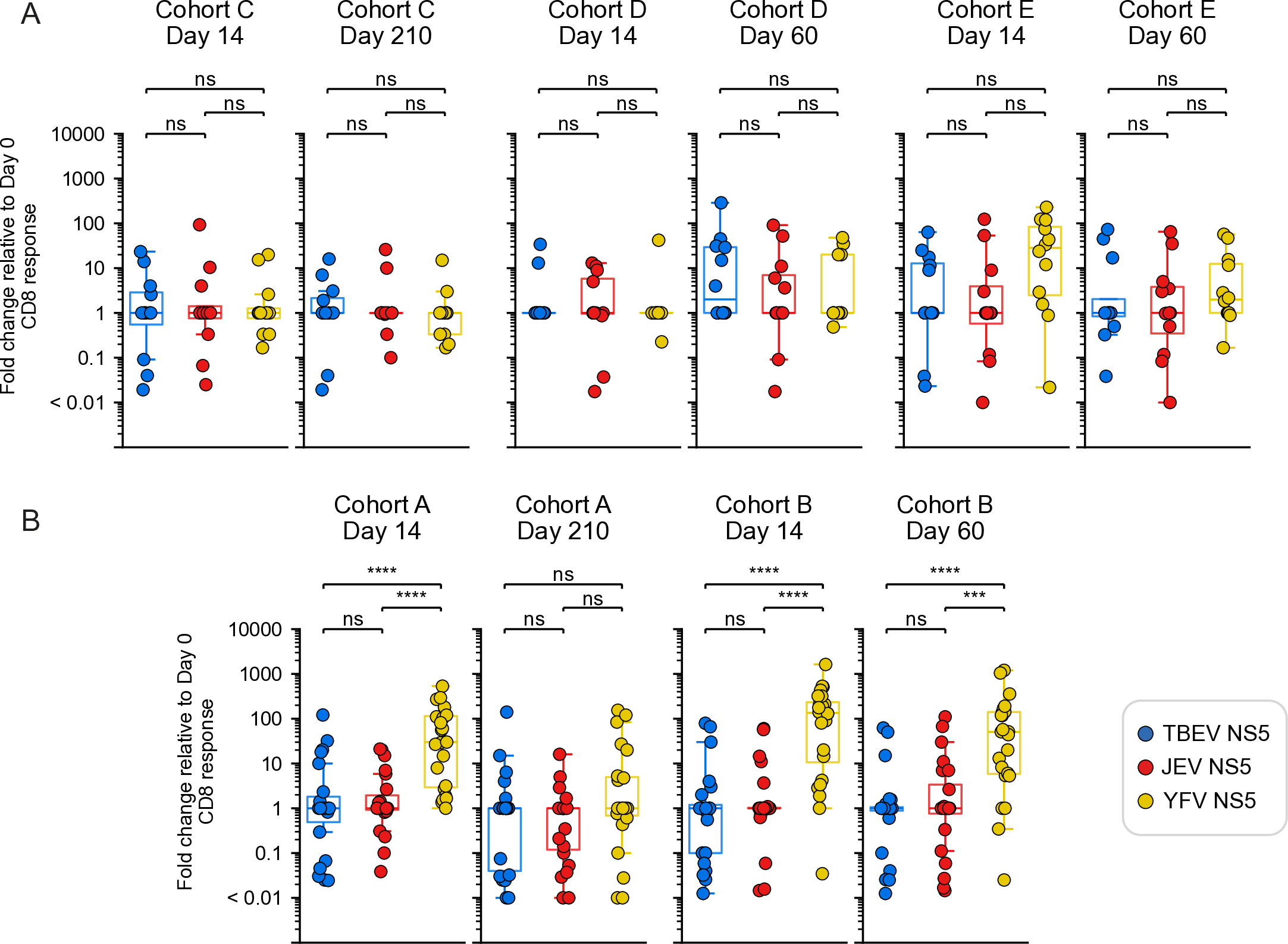

Supplement: S13 Fig — Comparisons of cross-reactive NS5-specific CD8+ T cell responses in the vaccine cohorts. (A-B) Frequency of TBEV NS5-specific CD8+ T cells in (A) cohorts C, D and E and (B) in cohorts A and B, expressed as fold change relative to day 0. (A-B) Statistical analysis assessed by Kruskal-Wallis’s and post hoc Dunn’s tests with Bonferroni correction. Distribution of data points is determined by boxplot with median and 25th-75th percentiles; whiskers are drawn from 1.5 times the IQR. Significant results are shown, where: *p ≤ 0.05, **p ≤ 0.01, ***p ≤ 0.001, ****p ≤ 0.0001. (TIF) [file pntd.0012693.s013.tif]

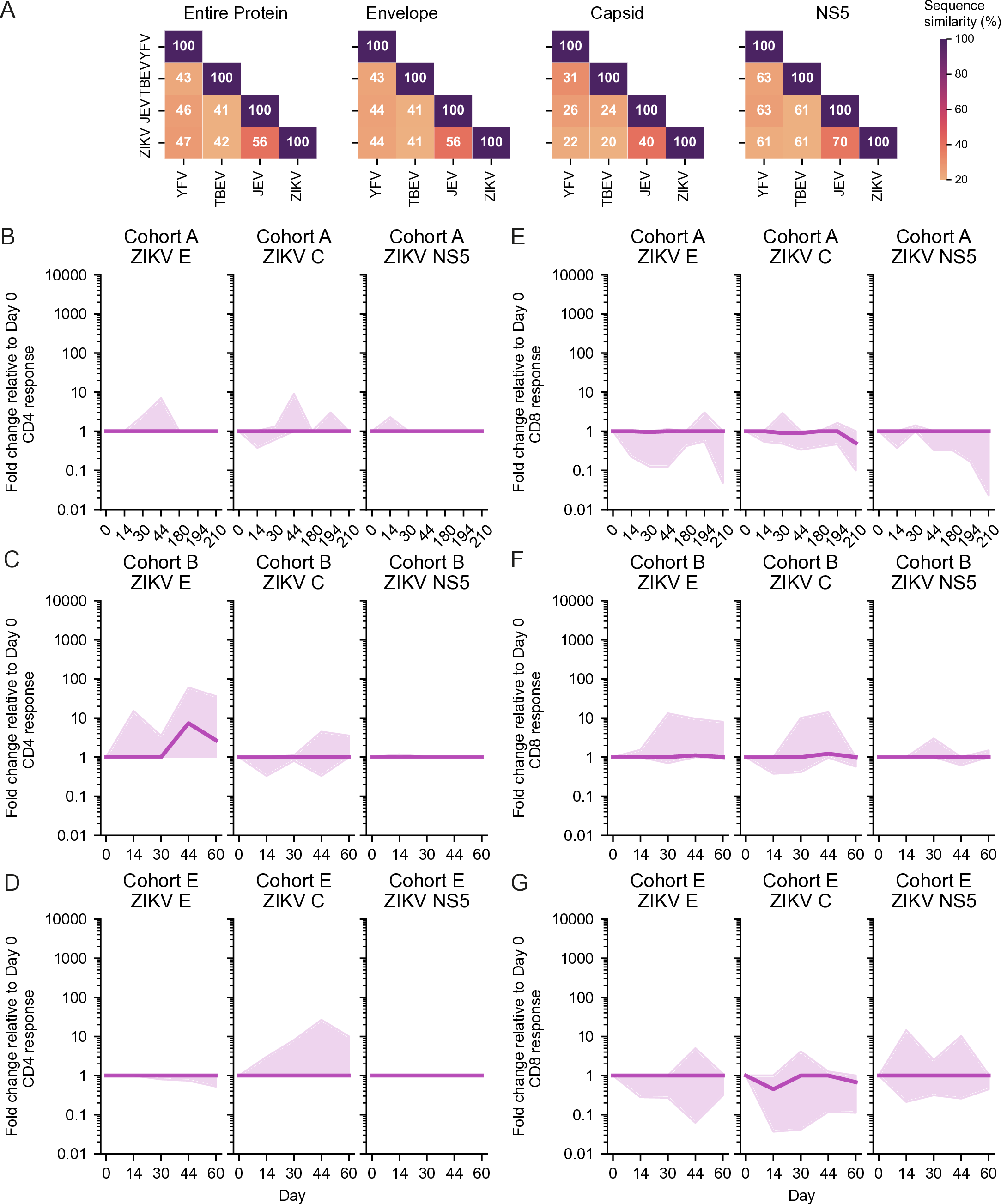

Supplement: S14 Fig — YFV vaccine-induced T cell response shows limited cross-reactivity with ZIKV antigens. (A) Sequence alignments between TBEV, JEV, YFV and ZIKV. Heatmaps are annotated according to percent of amino acid sequence similarities. (B) Frequency of ZIKV E, C- and NS5-specific CD4+ T cells in cohort A expressed as fold change relative to day 0. (C) Frequency of ZIKV E, C- and NS5-specific CD4+ T cells in cohort B expressed as fold change relative to day 0. (D) Frequency of ZIKV E, C- and NS5-specific CD4+ T cells in cohort E expressed as fold change relative to day 0. (E) Frequency of ZIKV E, C- and NS5-specific CD8+ T cells in cohort A expressed as fold change relative to day 0. (F) Frequency of ZIKV E, C- and NS5-specific CD8+ T cells in cohort B expressed as fold change relative to day 0. (G) Frequency of ZIKV E, C- and NS5-specific CD8+ T cells in cohort E expressed as fold change relative to day 0. (B-G) Line plots show median and 95% confidence interval (shaded area). (TIF) [file pntd.0012693.s014.tif]
