# Supplementary material for: Antigen-specific T cell responses following single and co-administration of tick-borne encephalitis, Japanese encephalitis, and yellow fever virus vaccines: Results from an open-label, non-randomized clinical trial-cohort
Source: PLoS Negl Trop Dis. 2025 Feb 28;19(2):e0012693. doi: 10.1371/journal.pntd.0012693 (PMC11893121; doi:10.1371/journal.pntd.0012693)
Supplement: S1 Table — (PDF) [file pntd.0012693.s016.pdf]

Supplementary Table 1. Antibodies used for flow cytometry experiments.

Antibody	Conjugate	Clone	Dilution	Catalog number	Company
LIVE/DEAD™ Fixable Aqua Dead Cell Stain Kit			1:1666.7	L34957	ThermoFischer Scientific
CCR7	APC-Cy7	G043H7	1:50	353212	BioLegend
CD137 (41BB)	PE-Cy7	4B4-1	1:25	309818	BioLegend
CX3CR1	PE	2A9-1	1:50	341604	BioLegend
CD69	BV650	FN50	1:50	310934	BioLegend
CD45RA	BV570	HI100	1:200	304132	BioLegend
CD14	BV510	M5E2	1:100	301842	BioLegend
CD19	BV510	HIB19	1:100	302242	BioLegend
CD154 (CD40L)	BV421	24-31	1:25	310824	BioLegend
CD38	APC-R700	HIT2	1:50	564979	BD Biosciences
CD3	BUV805	UCHT1	1:50	612895	BD Biosciences
CD4	BUV496	SK3	1:25	612936	BD Biosciences
CD8	BUV395	RPA-T8	1:250	563795	BD Biosciences
CXCR5	BB515	RF8B2	1:100	564624	BD Biosciences
CD40 (unconjugated)	-	HB14	1:200	130-094-133	Miltenyi Biotec
